# Getting More Out of Less – A Quantitative Serological Screening Tool for Simultaneous Detection of Multiple Influenza A Hemagglutinin-Types in Chickens

**DOI:** 10.1371/journal.pone.0108043

**Published:** 2014-09-23

**Authors:** Gudrun S. Freidl, Erwin de Bruin, Janko van Beek, Johan Reimerink, Sjaak de Wit, Guus Koch, Lonneke Vervelde, Henk-Jan van den Ham, Marion P. G. Koopmans

**Affiliations:** 1 Department of Viroscience, Erasmus Medical Center, Rotterdam, The Netherlands; 2 Emerging Infectious Diseases, Division of Virology, Centre for Infectious Diseases Research, Diagnostics and Perinatal Screening, Centre for Infectious Disease Control, National Institute for Public Health and the Environment (RIVM), Bilthoven, The Netherlands; 3 GD Animal Health Service (AHS), Deventer, The Netherlands; 4 Central Veterinary Institute (CVI), Lelystad, The Netherlands; 5 Utrecht University, Utrecht, The Netherlands; The University of Tokyo, Japan

## Abstract

Current avian influenza surveillance in poultry primarily targets subtypes of interest for the veterinary sector (H5, H7). However, as virological and serological evidence suggest, surveillance of additional subtypes is important for public health as well as for the poultry industry. Therefore, we developed a protein microarray enabling simultaneous identification of antibodies directed against different HA-types of influenza A viruses in chickens. The assay successfully discriminated negative from experimentally and naturally infected, seropositive chickens. Sensitivity and specificity depended on the cut-off level used but ranged from 84.4% to 100% and 100%, respectively, for a cut off level of ≥1∶40, showing minimal cross reactivity. As this testing platform is also validated for the use in humans, it constitutes a surveillance tool that can be applied in human-animal interface studies.

## Introduction

Avian influenza A viruses (AIV) belong to the family *Orthomyxoviridae* and comprise eight gene segments consisting of negative sense single-stranded RNA. The classification of AIV into different subtypes is based on two surface structures, hemagglutinin (HA) and neuraminidase (NA). To date, 18 distinct HA-types and 11 NA-types are known of [1]–[3]. With the exception of subtypes H17N10 and H18N11 of which RNA was recently detected in bats, aquatic birds constitute reservoirs for AIV, usually without showing signs of disease [1], [2]. To date, influenza A viruses have crossed the species barrier to humans, swine, aquatic mammals, domestic poultry, birds of prey, horses, mustelids, civets, felines and canines [4]–[6]. Several avian and swine influenza viruses have zoonotic potential. While AIV subtype A (H5N1) virus infections have had the largest economic and public health impact so far, AIV with HA types 6, 7, 9 and 10 have also caused virologically confirmed human infection with varying severity [4], [6]. Until recently, human H7-infections have been associated with mild symptoms in humans. However, since early 2013, a newly emerging H7-subtype, A(H7N9), has formed an exception by causing a more severe clinical picture and death in about 36% of the recorded patients, possibly related to specific host susceptibility factors [7], [8]. Although the symptoms shown by patients largely resembled infection with highly pathogenic (HP) A(H5N1), the manifestation in poultry – the putative source of direct human infection – is different [9], [10]. Unlike HP A(H5N1) viruses that cause severe illness and death in poultry, this novel influenza A(H7N9) strain causes subclinical infection in poultry, which allowed the virus to spread unnoticed over a large geographic region in China [10]. Consequently, the general population can be exposed to animals shedding this virus without warning signs. Indeed, serological investigations in poultry workers suggest more widespread infections in humans, possibly reflecting mild or unapparent illness [11].

This example and additional serological evidence for human infection with influenza viruses *other than* H5, H7, H9 and H10 – including H4, H6 and H11 [12]–[14] – highlight the importance of influenza monitoring at the human-animal interface, where humans are currently sentinels for circulation of zoonotic viruses [15], [16]. Therefore, ideally, future serological studies evaluating influenza viruses at the human-animal interface would include these “neglected” subtypes.

Given the ability of AIV H5 and H7 to mutate into HP forms and the economic consequences associated with such infections, a compulsory European Union-wide surveillance system was implemented in 2005 [17]. In the Netherlands, serological monitoring is more intensive than required by EU-regulations [18] and includes screening of all poultry flocks at least once a year and high risk-groups, e.g. free-range flocks every three months. In practice, a representative number of farms and individuals per country are pre-screened with an indirect or competitive enzyme-linked immunosorbent assay (ELISA), identifying antibodies against conserved regions (matrix or nucleoprotein) that all influenza virus subtypes have in common [19]. Upon a positive pre-screening result, the presence of H5- or H7-antibodies is confirmed or ruled out by means of a hemagglutination inhibition (HI)-assay, and flocks are tested for active virus circulation. While this screening regimen meets the requirements for veterinary surveillance, the characterization of non-H5 and -H7 but ELISA-positive samples may be relevant for the poultry industry and for public health.

Here, we describe the development and use of a protein microarray (PA) that enables simultaneous screening for antibodies to multiple influenza HA-types in poultry, using minute quantities of serum (10 µl) that can be collected through routine veterinary surveillance.

## Materials and Methods

### Sera

Three different serum sets (hereafter referred to as group 1–3) were used to evaluate the performance of the PA for the use in chicken:

1. **Negative sera.** Negative sera were obtained from different sources. In total 38 chicken sera which tested negative by ELISA (Idexx FlockChek AI, MultiS-Screen Ab Test Kit, Hoofddorp, the Netherlands) were used:
  1a) One serum pool of 52-week-old, specific pathogen free (SPF) white layers (flock from GD AHS)
  1b) Ten sera from 3-week-old, non-infected, non-vaccinated, conventional Lohman Brown layers
  1c) 27 sera from a commercial 6-week-old Ross broiler flock (hereafter named “negative field chickens”)
2. **Consecutive serum samples from SPF chickens experimentally infected with live field strains.**
  Four groups of 15 white SPF laying hens (GD AHS) were intratracheally infected with live field strains (0.5 ml; ∼10^6^ EID_50_) belonging to the subtypes H5N2, H6N2, H7N1 or H9N2 (Table 1) at 12 weeks of age. For the duration of the experiment infected chickens were kept in isolators with twelve hours light per day, 20–25°C and were given ad libitum access to food and water. Serum was collected from the wing vein at day 7, 14 and 22 post infection (p.i.) and seropositivity was confirmed by testing sera at one dilution (1∶8) by standard HI-assay, as is done routinely in the animal health service. Therefore, data were available as positive/negative results only.

**Table 1:** Recombinant HA1-proteins used on the PA and viruses used for infection of chickens of group 2.

Proteins	Subtype	Strain	Source
H1.18	H1N1	A/South Carolina/1/18	IT
H1.33	H1N1	A/WS/33	IT
H1.99	H1N1	A/New Caledonia/20/99	IT
H1.07	H1N1	A/Brisbane/59/2007	IT
H1.09	H1N1	A/California/6/2009	IT
H2.05	H2N2	A/Canada/720/05	IT
H3.68	H3N2	A/Aichi/2/1968	SB
H3.03	H3N2	A/Wyoming/3/03	IT
H4.02	H4N6	A/mallard/Ohio/657/2002	E
H5.97	H5N1	A/Hong Kong/156/97 (clade 0)	IT
H5.06	H5N1	A/Turkey/15/2006 (clade 2.2)	G
H5.02	H5N8	A/duck/NY/191255-59/2002 (LP)	SB
H5.07	H5N3	A/duck/Hokkaido/167/2007 (LP)	SB
H6.07	H6N1	A/northern shoveler/California/HKWF115/2007	SB
H7.03	H7N7	A/Chicken/Netherlands/1/03	IT
H8.79	H8N4	A/pintail duck/Alberta/114/1979	E
H9.99	H9N2	A/Guinea fowl/Hong Kong/WF10/99	IT
H9.07	H9N2	A/Chicken/Yunnan/YA114/2007	G
H11.02	H11N2	A/duck/Yangzhou/906/2002	IT
H12.91	H12N5	A/green-winged teal/ALB/199/1991	IT
H13.00	H13N8	A/black-headed gull/Netherlands/1/00	IT
H16.99	H16N3	A/black-headed gull/Sweden/5/99	IT
			

Infection	Subtype	Strain	GISAID accession number
	H5N2	A/chicken/Belgium/150/1999	EPI238402
	H6N2	A/turkey/Massachusetts/3740/1965	EPI3187
	H7N1	A/parrot/Northern Ireland/VF-73-67/73	EPI6514
	H9N2	A/chicken/Saudi Arabia/SP02525/3AAV/2000	AHS

LP, low pathogenic; IT, Immune Technology Corp.; SB, Sino Biological Inc.; E, e-enzyme; G, Genscript; AHS, from Animal Health Service, Deventer, the Netherlands.

3. **Sera from outbreaks of avian influenza detected during routine surveillance in the Netherlands.**
  To evaluate applicability of the test in the field, we analyzed samples from four different laying hen flocks having undergone past infection with low pathogenic (LP) AIV subtypes, hereafter named “naturally infected field chickens”. All flocks were identified as AIV exposed by ELISA-testing (Idexx FlockChek AI, MultiS-Screen Ab Test Kit) of samples collected during routine surveillance performed by the AHS. HI typing of sera, and/or virus isolation and virus typing (CVI, Lelystad) confirmed initial diagnosis. Samples were derived from two outbreaks caused by subtype H6, both in flocks of 16-month-old, free-range brown laying hens (outbreak 1: n = 10; outbreak 2: n = 7). In addition, ten sera seropositive for LP H7N3 were obtained from 16-month-old, free-range brown layers, and eight sera from an H9N2-outbreak in 19-month-old, brown laying hens housed in cages were screened. Individual HI-titers were available for the H9- and one H6-outbreak (outbreak 2). Sera of the remaining two outbreaks were screened qualitatively at one dilution only (1∶8).

### Ethics statement

All experiments were approved by the Animal Experimental Committee of the Faculty of Veterinary Medicine of the Utrecht University or the Animal Welfare Committee (DEC) of the GD Animal Health Service, Deventer, the Netherlands, in accordance with the Dutch regulations on experimental animals.

### Production of protein microarray-slides and sample analysis

We used a modification of the technique that has been described elsewhere [20]. In our study, 22 recombinant HA1-proteins comprising representatives of 13 different subtypes (Table 1) were printed onto 16-pad nitrocellulose slides as described before [20]. Antigens were produced in human embryonic kidney (HEK) cells, were purified by HIS-tag and were delivered at a protein concentration of 1 mg/ml (see manufacturer for details, Table 2). To determine the optimal working concentration for the recombinant HA1-proteins used in the PA, checkerboard titrations were performed for each protein using four different dilutions (2×, 4×, 8×, 16×). When necessary, proteins were concentrated using Amicon Ultra-0.5 mL Centrifugal Filters for Protein Purification and Concentration according to manufacturer's instructions (Merck Millipore, Massachusetts, USA) and checkerboard titrations were repeated thereafter.

**Table 2:** Sensitivities (%) for microarray antigens corresponding to subtype of virus strains used for infection of SPF chickens (group 2) according to time point of serum collection and different cut of levels. Bold font indicates 100% sensitivity.

	Virus subtype	H5				H6	H7	H9	
p.i.	Cut off ≥	H5.97	H5.06	H5.02	H5.07	H6.07	H7.03	H9.99	H9.07
Day 7	1∶20	**100.0**	**100.0**	85.7	**100.0**	**100.0**	80.0	86.7	73.3
	1∶40	92.9	92.9	78.6	**100.0**	**100.0**	73.3	73.3	66.7
	1∶80	85.7	85.7	57.1	85.7	**100.0**	66.7	20.0	26.7
	1∶160	78.6	78.6	28.6	57.1	**100.0**	53.3	6.7	13.3
	1∶320	50.0	57.1	21.4	0.0	93.3	40.0	0.0	0.0
	1∶640	14.3	28.6	0.0	0.0	86.7	6.7	0.0	0.0
	1∶1280	0.0	0.0	0.0	0.0	53.3	0.0	0.0	0.0
	1∶2560	0.0	0.0	0.0	0.0	13.3	0.0	0.0	0.0
Day 14	1∶20	**100.0**	**100.0**	**100.0**	**100.0**	**100.0**	**100.0**	**100.0**	93.3
	1∶40	**100.0**	**100.0**	85.7	**100.0**	**100.0**	**100.0**	93.3	86.7
	1∶80	92.9	92.9	78.6	**100.0**	**100.0**	**100.0**	80.0	86.7
	1∶160	85.7	78.6	57.1	**100.0**	**100.0**	93.3	20.0	46.7
	1∶320	78.6	71.4	42.9	71.4	**100.0**	66.7	13.3	26.7
	1∶640	71.4	50.0	14.3	28.6	**100.0**	60.0	6.7	20.0
	1∶1280	21.4	14.3	0.0	14.3	80.0	0.0	0.0	6.7
	1∶2560	7.1	7.1	0.0	0.0	40.0	0.0	0.0	0.0
Day 22	1∶20	**100.0**	**100.0**	**100.0**	**100.0**	**100.0**	**100.0**	**100.0**	**100.0**
	1∶40	**100.0**	**100.0**	**100.0**	**100.0**	**100.0**	**100.0**	**100.0**	**100.0**
	1∶80	**100.0**	93.3	**100.0**	**100.0**	**100.0**	**100.0**	**100.0**	93.3
	1∶160	**100.0**	86.7	**100.0**	**100.0**	**100.0**	78.6	46.7	73.3
	1∶320	86.7	73.3	86.7	80.0	**100.0**	64.3	20.0	46.7
	1∶640	80.0	53.3	66.7	53.3	**100.0**	21.4	6.7	20.0
	1∶1280	73.3	40.0	26.7	26.7	73.3	0.0	0.0	6.7
	1∶2560	13.3	0.0	6.7	13.3	40.0	0.0	0.0	0.0
Days combined	1∶20	**100.0**	**100.0**	95.3	**100.0**	**100.0**	93.2	95.6	88.9
	1∶40	97.7	97.7	88.4	**100.0**	**100.0**	90.9	88.9	84.4
	1∶80	93.0	90.7	79.1	96.6	**100.0**	88.6	66.7	68.9
	1∶160	88.4	81.4	62.8	89.7	**100.0**	75.0	24.4	44.4
	1∶320	72.1	67.4	51.2	58.6	97.8	56.8	11.1	24.4
	1∶640	55.8	44.2	27.9	34.5	95.6	29.5	4.4	13.3
	1∶1280	32.6	18.6	9.3	17.2	68.9	0.0	0.0	4.4
	1∶2560	7.0	2.3	2.3	6.9	31.1	0.0	0.0	0.0

p.i., post infection.

Prior to testing, all sera were inactivated in a water bath at 56°C for one hour. For serum analysis, four slides fixed in a FAST frame slide holder (Whatman, Kent, UK) could be used simultaneously. Each holder accommodated up to seven sera and one in house-standard. Serum was titrated in two fold dilution series ranging from 1∶20 (10 µl of serum) to1∶2560. Known negative sera were tested in two-fold dilutions ranging from 1∶20 to 1∶160. An in house-standard, comprising of a serum-pool of hyperimmunized chickens infected with strains of subtypes H5, H6, H7 and H9 was included in each test run. After serum incubation, bound antibodies were visualized using a Cy5 AffiniPure rabbit anti-chicken IgY Fc-fragment-specific conjugate (Jackson ImmunoResearch, West Grove, USA) diluted in Blotto Blocking Buffer (Thermo Fisher Scientific Inc., Rockford, MA, USA) and 0.1% Surfact-Amps (Thermo Fisher Scientific Inc.) at a concentration of 1∶1300. IgY represents the avian equivalent of mammalian IgG [21].

### Data analysis and statistics

Fluorescent signals were quantified and converted into titers as described before [20]. The PA spanned a detection range of titers from 1∶20 to 1∶2560. We calculated geometric mean titers (GMTs) including 95% confidence intervals (CI) as well as homologous versus heterologous GMT ratios of the validation data using GraphPad Prism for Windows (Version 6.03, GraphPad Software Inc., California, USA). Log2-transformed median antibody titer ratios of field chickens were plotted in R (R Foundation for Statistical Computing, version 2.15). For consecutively collected samples, seroconversion or a significant rise was defined as a ≥4 fold increase in antibody titer [22]. Correlations between the PA and HI-test were calculated using a two-sided Spearman's rank correlation coefficient (ρ). A p-value of less than 0.05 was considered statistically significant.

The overall antibody reactivity for all seropositive individuals was visualized by means of a heat map, generated by applying hierarchical clustering (pairwise correlation distance and Ward's method) to log-transformed titers. No cut off titer was applied to the data. Bright red color indicates high titers whereas faint red and white corresponds to low titers and no reactivity, respectively. Amino acid (AA) sequence similarity of HA1s was determined using a fast algorithm with pairwise alignment in Bionumerics (version 6.6, Applied Maths).

### Antigen stability and batch control

Antigen quality and stability between different batches was tested using an in-house serum pool comprising HA-specific polyclonal rabbit-antisera (Immune Technology Corp., New York, USA) raised against all antigens included on the PA. Testing the last slide from each batch of 25 slides showed that all antigens were stable over time (data not shown). Prior experiments showed that spotted PA slides containing recombinant influenza HA1-proteins are stable for at least one year (unpublished data). Day-to-day variation was controlled for by correcting all titers according to the reactivity of the reference antigen H6.07 against the in house-standard, as previously described [20].

## Results

Four out of 38 negative sera (1.5%) – all four belonging to group 1c (negative field chickens) – showed minor low-level reactivity with titers ranging between 21 and 30 against antigens H2.05 and H12.91, respectively. All other samples tested negative for all antigens (data not shown). These findings result in a specificity of the PA of 94.6% to 100% at a cutoff titer of>1∶20 across all antigens, and of 100% when the cutoff was raised to ≥1∶40 or higher.

**Figure 1:**
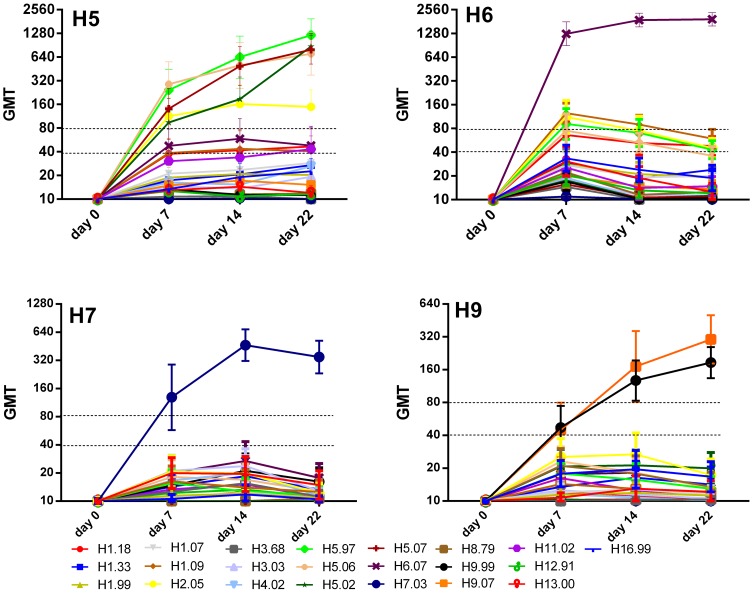
Kinetics of serological responses of SPF chickens after intratracheal infection with live virus (group 2). Titles of each graph indicate infection group. X-axes depict the day of serum collection post infection. Y-axes indicate geometric mean titers (GMT). Error bars represent 95% confidence intervals of the measurements. Note differences in log-scale. Heterologous reactions above the dotted line represent cross-reactive responses with a titer higher than 1∶40 or 1∶80, respectively.

In contrast, all experimentally infected chickens (group 2) seroconverted to the homologous antigens, although the kinetics of response differed slightly. H5- and H6-infected animals were the fastest to show 100% seroconversion at day 7 p.i. at a cut off of ≥1∶40 for at least one PA-antigen used, whereas for H7- and H9-exposed animals complete seroconversion (100% of animals) occurred at a later time point (Figure 1, Table 2). At day 22 p.i. all animals showed a significant (≥ 4-fold) titer increase. With advancing antibody rise (at days 14 and 22 p.i.), sensitivities further increased for antigens matching the infecting subtype. In addition, we combined all serum collection time points to investigate the ability of the PA to identify positive individuals in different stages of antibody development and sensitivity remained high (Table 2).

Interestingly, although H5-infected SPF chickens were inoculated with a low-pathogenic H5-strain (Table 1), we observed the strongest antibody response against H5.97, an antigen representing HP AIV clade 0 (Table 1, Figure 1). For the H9-infection cohort, chickens showed mixed antibody reactivity against the two H9-antigens, with half the individuals reacting stronger against H9.99 and the other half displaying a higher titer against H9.07 at day 7 p.i. One individual had an equally high titer for both antigens at that time point. At day 14 and 22 p.i., reactivity profiles shifted towards H9.07 in the majority of chickens, ten and nine out of 15, respectively, displaying a higher titer against H9.07 compared to H9.99 (data not shown).

### Cross-reactivity against heterologous antigens of experimentally infected chickens (group 2)

**Figure 2:**
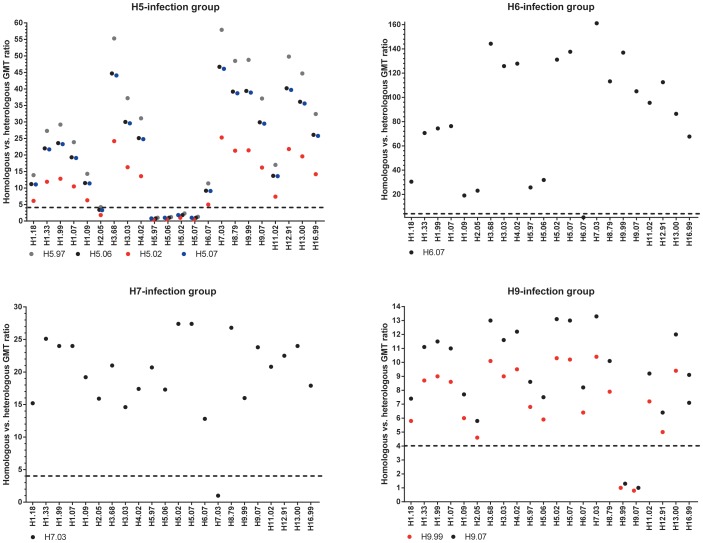
Homologous versus heterologous geometric mean titer (GMT) ratios for different groups of experimentally infected chickens (H5, H6, H7, H9) for all sampling days combined. A high homologous versus heterologous ratio in GMT indicates low cross-reacitivity and vice versa. For instance, as for the H6-infection group the GMT against the homologous antigen H6.07 is 1668.8 and the GMT against the heterologous antigen H7.03 is 10.4, the homologous vs. heterologous ratio is the highest (∼161), implying that the level of cross-reactivity is lowest for the H7-antigen in the H6-infection group. The dotted line demarkates a ratio of 4. Note differences in scale.

In general, we observed some degree of heterogeneity in kinetics and cross reactivity of antibody responses within all infection groups (Figure 1). The ratio of homologous versus heterologous GMTs of all sampling days combined ranged from 1.8 to 57.9 in H5-, 19.1 to 161.1 in H6-, versus 12.8 to 27.4 for H7- and 4.6 to 13.3 in H9-infected individuals (Figures 1 and 2). The highest level of cross reactivity was observed in H5-infected animals reacting with the H2-antigen (GMT-ratio 1.8-4.2). Nevertheless, a clear distinction between homologous and heterologous reactivity was observed for the remaining antigens, with GMT ratios of >4 for all other antigen combinations (Figures 1 and 2). Therefore, the infecting strain could clearly be identified independent of the cutoff level chosen (Figure 1). To minimize or dismiss the “noise” caused by cross-reacting antibodies, the application of a cutoff level of ≥1∶80 seems appropriate (Table 2, Figure 1).

### Serological profiles of naturally infected laying hens (group 3)

**Figure 3:**
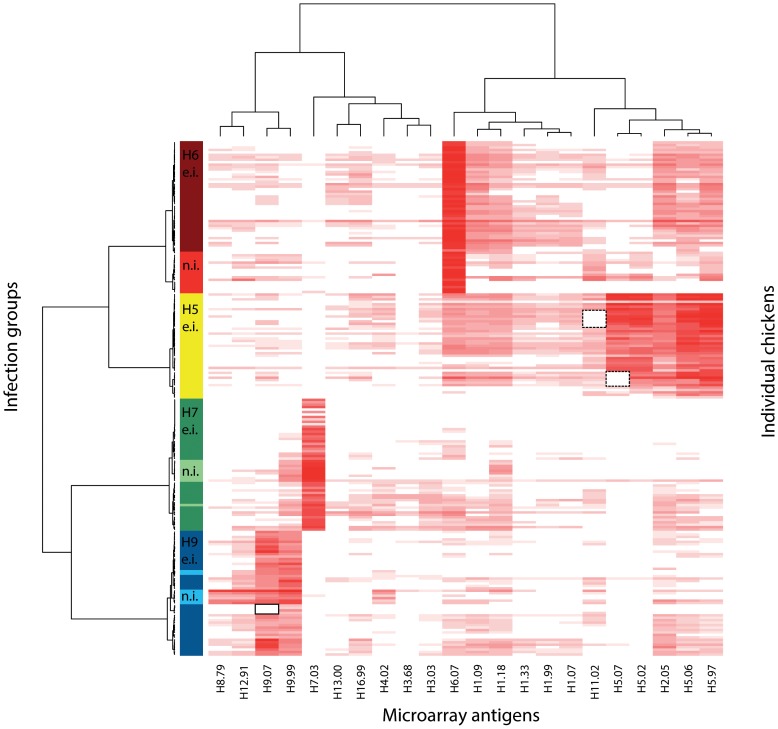
Heat map depicting serological patterns of naturally (n.i.) and experimentally infected (e.i.) chickens (H5, H6, H7, H9) spanning all samplings days. Dendrograms reflect clustering based on similarity of serological profiles. Microarray antigens are depicted on the X- axis. Different infection groups are color coded on the Y-axis according to the avian influenza virus subtype causing the infection. Rows represent reaction profiles of individual chickens across the entire antigen panel. Columns represent the reactivity of all individuals against a specific antigen as stated on the X-axis. Intensity of the red color is proportional to the log-titer height. Black dotted squares indicate missing antigens H5.07 (n = 6) and H11.02 (n = 7) due to spotting failure. Black square with solid line indicates no biological reactivity against the H9.07 antigen. The clustering algorithm automatically excluded negative sera.

**Figure 4:**
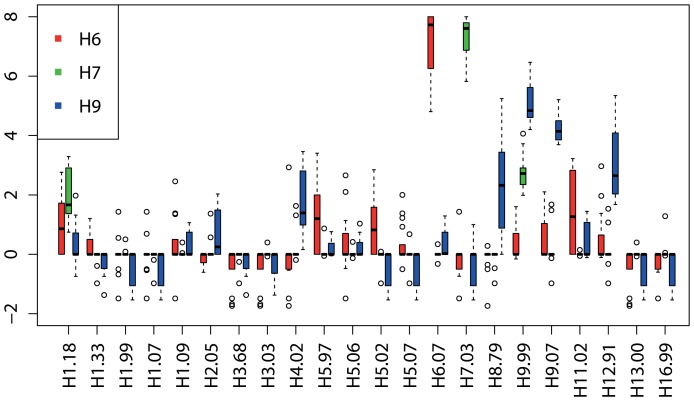
Antibody profiles of field chickens expressed as log2-transformed median antibody titer ratios plotted per outbreak. Antibody titer ratios were derived by log transforming the data, calculating the median antibody reactivity across all antigens included on the PA and subtracting it from the antibody reactivity against individual antigens. This was calculated for every chicken. By doing that, every individual's values are normalized according to its own background reactivity. Individual ratios were summarized in boxplots. Horizontal bars within each box represent log2-transformed median antibody titer ratios per antigen and outbreak. Chickens naturally infected with H6 are depicted in red (n = 17), H7 in green (n = 10) and H9 in blue (n = 8). The two H6-outbreaks were combined in this analysis.

Serum samples from naturally infected field chickens showed similar discriminatory serological profiles compared with the data from the validation experiments (Figure 3). In the analysis, we combined the data of both H6-outbreaks. The PA correctly identified 100% of the tested field chickens as positive up to a cut-off titer of ≥1∶80 (data not shown). Cross-reactivity was negligible for H6- and H7-infected individuals and generally matched the patterns observed in group 2 (Figure 3, light red, light green). Among the field chickens naturally infected with H9, we observed somewhat more cross reactivity (Figure 3 and 4). Nevertheless, the infecting subtype was still evident by resulting in the highest median log2-titer ratio (Figure 4).

Overall, the PA results showed good correlation with the HI-assay. Spearman's rank correlation coefficients showed strong, significant associations between the HI-assay and PA-antigens H9.07 (ρ = 0.804, p = 0.021) and H6.07 (ρ = 0.850, p = 0.029), whereas a relatively strong but not statistically significant association could be demonstrated between HI-data and PA-antigen H9.99 (ρ = 0.600, p = 0.121).

## Discussion

Here we present a highly sensitive and specific multiplex-screening tool to detect antibodies against different HA-types of AIV in chickens. We show that the PA discriminates between negative and experimentally infected, seropositive chickens. We further demonstrate that our test can serve as a surveillance tool in commercial field chicken flocks, by reliably identifying the infecting subtypes in laying hens from free-range- and indoor husbandry. An asset of the technique is that it requires a minute quantity of serum (5–10 µl) to simultaneously screen for *multiple* subtypes, whereas the HI-assay usually requires about the same amount to detect antibodies against only *one* subtype [23]. This characteristic is particularly advantageous when screening small animal species of which only small volumes of sera are available.

Analysis of consecutive sera of SPF chickens infected with live field strains of different AIV subtypes showed that the PA was able to quantify varying titer heights per sampling time point and infection group. Such variation could either be due to differences in immunogenicity of strains used for infection [24], infectious dose [24], [25], different chicken breeds or genetic lineages [24], [26]. From a technical aspect, differing quality of antigens used on the PA and distant relatedness of strains used for infection and the assay antigen [27] could account for the differences in titer heights between infection groups. As infectious dose and breed were the same for all experimental infection cohorts and antigen quality was checked prior to testing and monitored throughout the experiment, these factors can be disregarded as a possible source of variability.

Lee et al. [28] speculated that immunogenicity, and therefore antibody titer heights, can depend on the protein itself and can vary between strains of different subtypes in chickens immunized with different DNA-vaccines. Failure to regularly update antigens in HI-assay can result in a reduced ability to detect antibodies against more recent field isolates [27] and it is unclear if this also can be observed in our assay system. The strains used for the infections of group 2 animals were closely related to the strains from which antigens were produced, with the lowest level of AA-identity for antigen H9.07 (94.4%) (Table 3). This lower AA-identity in combination with individual variation could be a possible explanation why H9.07 did not yet react at day 7 p.i. for some experimentally infected chickens of group 2 (Figure 3, square with black solid line). On the other hand, the lower AA-identity of H9.07 did not seem to have a major influence, as this antigen showed a higher GMT at day 14 and 22 p.i. in H9-infected chickens of group 2, compared to H9.99, which was 97.5% similar to the infecting strain (Table 3). It is not known how AA differences in HA1 translate to antigenic reactivity in the PA system. Cattoli et al. [29] examined serological responses of drift variants of H5 strains in chickens using HI- and microneutralization assay. Of the 11 AA substitutions found in the HA1, the researchers demonstrated that only five substitutions sufficed to cause antigenic drift. These findings stress that a high AA sequence similarity in the HA1 of two strains does not necessarily translate into similar serological reactivity, if critical substitutions occur in epitopes influencing antigenicity. Hence, AA sequence similarity is not a good indicator for antigenicity and cross reactivity, so no inferences about the compatibility between the viruses used for infection and PA-antigens can be made.

**Table 3:** Amino acid (AA) similarity matrix for strains of a particular subtype used to infect SPF chickens of group 2 (in bold) versus PA-antigens per HA-type (table 1).

**H5N2**	***100.00***				
*H5.07*	***97.37***	100.00			
*H5.02*	***96.15***	96.42	100.00		
*H5.97*	***95.75***	95.39	93.52	100.00	
*H5.06*	***95.29***	95.45	94.21	97.48	100.00
**H6N2**	***100.00***				
*H6.07*	***96.01***	100.00			
**H7N1**	***100***				
*H7.03*	***97.11***	100.00			
**H9N2**	***100.00***				
*H9.99*	***97.49***	100.00			
*H9.07*	***94.39***	93.77	100.00		

Similarity was calculated based on the HA1 part of the hemagglutinin (sequence length 318 AA). Percentages in bold and italics denote similarity between strains used for infection versus corresponding PA-antigen.

Overall, the observed cross-reactivities were negligible in comparison to the titer height of the antigens matching the subtype of infection. Interestingly, we noted that heterologous patterns largely reflected phylogenic relationships. The 16 currently known HA-types derived from birds divide into two phylogenetic groups which further segregate into 5 clades. Group 1 consists of 3 clades (H1, H2, H5 and H6; H8, H9 and H12; H11, H13 and H16) whereas group 2 comprises 2 clades (H3, H4 and H14; H7, H10 and H15) [1], [30]. Heterosubtypic immunity has mainly been attributed to cytotoxic T-cells specific for internal proteins [31], but neutralizing antibodies also play an important role in protection [32], [33]. To date, a number of broad reacting intra-subtype-, intra-clade-, intra-group- and inter-group-specific neutralizing monoclonal antibodies have been identified [34]–[37]. Of all vaccination cohorts, H5-vaccinated chickens displayed the highest level of cross-reactivity with antigen H2.05 (Figures 1, 2 and 3). This finding is not surprising due to the high sequence similarity of these two subtypes [28], [38]. Likewise, H9-positive serum cross-reacted somewhat with members of the same clade, H8 and H12. Together with the calculation of the median-log2-titer ratios – as was performed for the field chickens in this study –, the knowledge of these patterns can be useful in distinguishing cross-reactivity from potential dual infections involving subtypes of different clades. Although we only tested one serum of a chicken simultaneously immunized with influenza virus strains belonging to two different subtypes (H7 and H9), the PA showed clear antibody titers against both HA-types (median log2-titer ratio for H7.03 = 8, H9.99 = 6 and H9.07 = 5.8, respectively) with no cross-reactivity to other antigens (median log2-titer ratio  = 0). This capacity can be especially interesting for regions where AI surveillance is not implemented in poultry and where animals might experience multiple consecutive- or co-infections with different subtypes. To further investigate this potential, serum of experimentally infected chickens consecutively or simultaneously immunized with different subtypes would need to be analyzed, which were not available in this study.

Heterologous reaction was lowest in chickens experimentally and naturally infected with subtype H7 compared to other serum cohorts. This can possibly be explained by the fact that, apart from H3- and H4-antigens, no other representatives of phylogenetic group 2 (H10, H14, H15) were included in the PA setup. Similarly, Latorre-Margaleff et al. [39] found that after infection with a certain subtype, infection with the homologous- or subtypes within the same clade and group were uncommon, suggesting heterosubtypic immunity.

In this project, we showed that the PA can discriminate between different HA-types. Strain-discrimination was not possible yet with the PA, when more than one antigen per subtype was included, e.g., H5. This intra-subtype reactivity is not unexpected since a study found an intra-subtype similarity (based on AA-sequences of the HA1) of >92%, whereas inter-subtype identity based on AA-similarity was much lower (38.5%) [40]. Broad intra-subtype reactivity is exploited in diagnostics. Ducatez et al. [41] discovered that ancestral strain A of HP H5N1 as well as strains belonging to clade 2.2 (represented by H5.06 in our study) proved to be the most suitable antigen as they correctly identified most HP H5N1 antigens/-sera of other clades [41]. On the other hand, as genetic changes can lead to escape mutants eliciting different serological responses, it is important to monitor and regularly update the PA-antigen setup, as is done for other serological assays [27]. The extent to which strain discrimination can be achieved by means of the PA is currently focus of a follow up project.

It is important to stress that the PA does not give information on the presence or absence of neutralizing antibodies and can therefore not be used to determine the immune status, i.e. protection. In serological avian influenza surveillance the HI assay is currently the gold standard with a sensitivity and specificity of 98.8% and 99.5%, respectively [42]. Overall, the PA showed a good correlation with the HI test. Other currently known serological multiplex techniques for the use in poultry, e.g. bead-based Luminex assays, either target conserved regions of influenza virus (nucleoprotein, matrix protein, non-structural protein 1) [43], screen for antibodies against HA-types relevant for the poultry sector (H5 and H7) [44] or combine the two approaches, eg. nucleoprotein with H5 [45]. In addition, simultaneous serological screening for influenza virus in combination with other poultry diseases of economic importance (e.g., Newcastle Disease Virus, Infectious Bronchitis Virus, Infectious Bursal Disease Virus) are described in the literature [46], [47]. To our knowledge, the PA technique is the first to allow simultaneous detection of influenza virus antibodies against more than two HA-types in chickens.

In this study, we aimed at including the full range of HA-types known to be present in birds at the time. The dependence on commercial availability lead to the random assembly of antigens of Eurasian as well as North American lineages and failure to cover all AIV subtypes. It is known that strains descending from Eurasian and North American lineages of H5 and H7 differ antigenically, as is reflected in differing titer heights in serological assays [28]. Therefore, to achieve optimal results, the PA should ideally comprise antigens relevant and topical for the region in which the test is to be deployed. A limitation that should be acknowledged is that the PA has only been tested with sera of subtypes H5, H6, H7 and H9. To evaluate the performance against other subtypes, additional serum cohorts would need to be analyzed. Furthermore, the PA is limited to the detection of HA-type specific antibodies and cannot identify antibodies against the neuraminidase. It is not known as to what extent NA-specific antibodies influence reactivities against the HA-proteins (due to steric hindrance) in this testing platform [28].

In conclusion, we present a sensitive and specific test for the simultaneous detection of HA-type specific antibodies against different AIVs in chicken that requires very low amounts of serum. In combination with a screening-ELISA targeting antibodies against a conserved region of AIV, the PA can provide a valuable epidemiological surveillance tool to monitor dispersal of different subtypes. As this testing platform is also validated for the use in humans [20], [48] it lends itself for conducting exposure studies at the human-animal interface. Current research centers on the development of the PA for the use in swine.
